# Relationship between site of oesophageal cancer and areca chewing and smoking in Taiwan

**DOI:** 10.1038/sj.bjc.6601251

**Published:** 2003-09-30

**Authors:** M-T Wu, D-C Wu, H-K Hsu, E-L Kao, J-M Lee

**Affiliations:** 1 Graduate Institute of Occupational Safety and Health and Department of Occupational Medicine, Kaohsiung Medical University, Kaohsiung, Taiwan; 2 Department of Gastroenterology, Kaohsiung Medical University Hospital, Kaohsiung, Taiwan; 3 Department of Chest Surgery, Kaohsiung Veterans General Hospital, Kaohsiung, Taiwan; 4 Department of Chest Surgery, Kaohsiung Medical University Hospital, Kaohsiung, Taiwan; 5 Department of Surgery, National Taiwan University Hospital, Taipei, Taiwan

**Keywords:** oesophageal cancer, anatomic site, cigarette smoking, betel chewing

## Abstract

Among 309 male patients, those who had heavily consumed betel and tobacco were more likely than nonchewers (OR=2.91; 95% CI=1.36–6.25) and nonsmokers (OR=2.49; 95% CI=1.02–6.08) to develop cancer in the upper and middle third of the oesophagus, respectively; the effects of alcohol did not dominate in any third.

Our earlier study reported that habitual substance uses, including cigarettes, alcohol and areca, were the major risk factors for developing oesophageal squamous cell carcinoma in Taiwan (Wu *et al*, 2001). Although much research has investigated oesophageal cancer risk factors (Nandakumar *et al*, 1996; Phukan *et al*, 2001; Wu *et al*, 2001), only one study, to our knowledge, from India has examined the association between substance use and the anatomic site of oesophageal cancer lesions (Nandakumar *et al*, 1996). We therefore attempted to clarify the influence of substance use on the anatomical site of oesophageal cancer occurrence in Taiwan.

## MATERIALS AND METHODS

### Selection of subjects

Over 6 years (1996–2002), we recruited 309 Taiwanese male patients with pathologically proved oesophageal squamous cell carcinoma from the National Taiwan University Hospital (Taipei), Kaohsiung Medical University Hospital and Kaohsiung Veterans General Hospital (Kaohsiung).

Subjects were interviewed by trained interviewers who collected demographic and substance use data by using a standardised questionnaire (Wu *et al*, 2001). This study was approved by Kaohsiung Medical University Hospital's IRB. Informed consent was obtained from all subjects. Information on habitual substance use included whether the subject had been a habitual areca chewer, cigarette smoker or alcoholic beverage drinker. Subjects who had smoked more than 10 cigarettes week^−1^ for at least 6 months were defined as cigarette smokers. Those who had regularly chewed betel quid for at least 6 months were defined as betel chewers. And those who had drunk beer, wine or distilled spirits more than one time week^−1^ for at least 6 months were defined as alcoholic beverage drinkers.

### Location and staging classification of oesophageal cancer

Lesions were classified with respect to their location in the upper, middle or lower third of the oesophagus (Rosenberg *et al*, 1981; Chalasani *et al*, 1998). Upper-third lesions extended from the cricopharyngeal sphincter (15 cm) to the tracheal bifurcation (23 cm). Middle-third lesions extended from 23 cm to the approximate level of the T9 vertebral body (32 cm). Lower-third lesions extended from 32 cm to the gastro-oesophageal junction (40 cm). If the lesion involved more than one-third, both locations were recorded. The American Joint Committee on Cancer (AJCC) staging system was used (AJCC, 1988).

### Statistical analysis

Unconditional logistic regression assessed the association between cancer location and substance use. Lesions in a given oesophageal third were compared to the remainder of the oesophagous.

For substance use, we used no cigarette smoking, no areca chewing and no alcohol consumption as baselines and compared these baselines to lifetime consumption. Users of substances were subdivided into two groups based on median levels of substance use. Lifetime consumption of tobacco was calculated by multiplying number of packs day^−1^ by the number of years smoked, yielding pack-years. Lifetime betel consumption was calculated by multiplying the average number of betel quids day^−1^ by the number of years chewed, yielding betel-years and alcohol by the number of years alcohol had been drunk (Wu *et al*, 2001). Variables in the models included age (>65 and ⩽65 years), educational level (⩾college, high school and ⩽elementary school) and substance use (tobacco, alcohol and areca). All *P*-values were two-sided.

## RESULTS

In total, 18.5% (57 out of 309), 41.7% (129 out of 309) and 26.5% (82 out of 309) of cancer lesions were located in the upper, middle or lower third of oesophagus, respectively (Table 1

**Table 1:** Demographic and clinical characteristics of oesophageal cancer (*n*=309)

**Variables**	***n* (%)**
Age (years)	
⩽65	184 (59.5)
>65	125 (40.5)
	
Educational levels	
⩽Primary school	184 (59.6)
High school	86 (27.8)
⩾College	39 (12.6)
	
Location of lesion	
U/3	57 (18.5)
M/3	129 (41.7)
L/3	82 (26.5)
U/3+M/3	11 (3.6)
M/3+L/3	30 (9.7)
	
Stage^a^	
Stage 0	2 (0.7)
Stage I	34 (12.5)
Stage IIA	51 (18.6)
Stage IIB	34 (12.4)
Stage III	111 (40.5)
Stage IV	42 (15.3)

U=upper; M=middle; L=lower.

a Missing=35.

); 3.6% (11 out of 309) and 9.7% (30 out of 309) of cancers involved both upper and middle thirds or middle and lower thirds of the oesophagus, respectively.

Median cutoff points for lifetime consumption of these substances were 35 pack-years for smokers, 400 betel-years for areca chewers (about 20 betel quid day^−1^ for 20 consecutive years) and 32 years for alcoholic beverage drinkers. After adjusting for age (⩽65 *vs* >65 years), educational level (⩽primary school *vs* ⩾college and high schools *vs* ⩾college) and other substance use (tobacco and alcohol), we found that compared to nonchewers, subjects who had more than a 400 betel-year history were 2.91-fold more likely to develop cancer in the upper third of the oesophagus (95% CI=1.36–6.25) (Table 2

**Table 2:** Relationship of substance use with site of oesophageal cancer (*n*=309)

	**Upper third**	**Non-upper third**		
**Variables**	***n* (%)**	***n* (%)**	**OR (95% CI)**	**AOR (95% CI)**
Cigarette smoking (pack-years)				
Nonsmokers	11 (14.5)	41 (15.9)	1.00	1.00
⩽35	30 (39.5)	114 (44.2)	1.29 (0.45–3.65)	1.31 (0.41–4.21)
>35	35 (46.0)	103 (39.9)	1.78 (0.64–5.00)	1.99 (0.63–6.24)
				
Areca chewing (betel-years)				
Nonchewers	43 (56.6)	162 (62.8)	1.00	1.00
⩽400	14 (18.4)	63 (24.4)	0.94 (0.47–1.87)	1.13 (0.52–2.44)
>400	19 (25.0)	33 (12.8)	2.40 (1.22–4.71)	2.91 (1.36–6.25)^a^
				
Alcohol consumption (years)				
Nondrinkers	20 (26.3)	58 (22.5)	1.00	1.00
⩽32	32 (42.1)	104 (40.3)	1.02 (0.49–2.13)	0.70 (0.28–1.76)
>32	24 (31.6)	96 (37.2)	0.86 (0.40–1.86)	0.57 (0.24–1.35)
				
	**Middle third**	**Non-middle third**	**OR (95% CI)**	**AOR (95% CI)**
**Variables**	***n* (%)**	***n* (%)**		
Cigarette smoking (pack-years)				
Nonsmokers	11 (6.5)	41 (14.4)	1.00	1.00
⩽35	79 (46.5)	62 (44.6)	2.32 (1.03–5.19)	2.22 (0.91–5.47)
>35	80 (47.0)	57 (41.0)	2.55 (1.14–5.74)	2.49 (1.02–6.08)^a^
				
Areca chewing (betel-years)				
Nonchewers	101 (59.4)	80 (57.6)	1.00	1.00
⩽400	44 (25.9)	32 (23.0)	1.09 (0.63–1.87)	0.79 (0.43–1.45)
>400	25 (14.7)	27 (19.4)	0.73 (0.40–1.36)	0.52 (0.26–1.02)
				
Alcohol consumption (years)				
Nondrinkers	27 (15.9)	30 (21.6)	1.00	1.00
⩽32	77 (45.3)	57 (41.0)	1.50 (0.81–2.80)	1.22 (0.57–2.64)
>32	66 (38.8)	52 (37.4)	1.41 (0.75–2.66)	1.16 (0.57–2.40)
				
	**Lower third**	**Non-lower third**	**OR (95% CI)**	**AOR (95% CI)**
**Variables**	***n* (%)**	***n* (%)**		
Cigarette smoking (pack-years)				
Nonsmokers	18 (16.1)	13 (6.6)	1.00	1.00
⩽35	50 (44.6)	91 (46.2)	0.40 (0.18–0.88)	0.40 (0.16–0.99)^a^
>35	44 (39.3)	93 (47.2)	0.34 (0.15–0.76)	0.31 (0.13–0.76)^a^
				
Areca chewing (betel-years)				
Nonchewers	70 (62.5)	111 (56.4)	1.00	1.00
⩽400	25 (22.3)	51 (25.9)	0.78 (0.44–1.37)	0.91 (0.48–1.71)
>400	17 (15.2)	35 (17.8)	0.77 (0.40–1.48)	0.89 (0.43–1.81)
				
Alcohol consumption (years)				
Nondrinkers	23 (20.5)	34 (17.3)	1.00	1.00
⩽32	43 (38.4)	91 (46.2)	0.70 (0.37–1.33)	0.96 (0.43–2.17)
>32	46 (41.1)	72 (36.5)	0.94 (0.50–1.80)	1.36 (0.64–2.90)

AOR=after adjusting for age (>65 *vs* ⩽65 years), educational level (⩽primary school, high school, ⩾college) and substance use (tobacco, alcohol and areca).

a *P*<0.01.

). In addition, we found that subjects who had smoked more than 35 pack-years were 2.49-fold more likely to develop cancer in the middle third of the oesophagus than were nonsmokers (95% CI=1.02–6.08). Results remained similar after adjusting for clinical stage (>Stage II *vs* ⩽Stage II) (data not shown). In contrast, smokers were less likely to develop cancer in the lower third of oesophagus than were nonsmokers. We found no significant effect of alcohol consumption on the location of oesophageal cancer.

## DISCUSSION

Very few studies have investigated the relationship between habitual substance use and location of oesophageal cancer (Nandakumar *et al*, 1996). Nandakumar *et al* studied oesophageal cancer (343 cases and 686 controls) in India and found that chewing areca preparations were associated with an increased risk for developing cancer in the middle third of the oesophagus. In contrast, chewing tobacco was associated with lesions in the lower third.

In Taiwan, tobacco is smoked, instead of being added to areca preparations for chewing. Therefore, we speculate that the inhalation of carcinogens from cigarette smoking indirectly affects the oesophagus after entering into the blood stream via pulmonary capillary absorption. Our results agree with those of Nandakumar *et al* (1996) who also found that cigarette smoking more likely affected the middle rather than upper third of the oesophagus.

On the other hand, the association that we found between areca chewing and oesophageal cancer in the upper third differs from the findings of Nandakumar *et al* (1996). Areca is chewed and sometimes the areca juice is swallowed. Therefore, besides the oral cavity and pharynx, the first contact area of areca juice is the upper third of the oesophagus. Since chewing areca can cause oral and pharyngeal cancers (Ko *et al*, 1995; Lee *et al*, 2003), our findings suggest that lesions in the upper third of the oesophagus might be due to direct mucosal exposure to areca preparation contents.

In summary, we found that chewing areca and smoking cigarettes were associated with lesions in the upper and middle thirds of the oesophagus, respectively. Further studies need to examine whether tissue from areca-associated oesophageal cancer is associated with elevated levels of areca-associated DNA adducts in the upper third of oesophagus. In addition, our findings need to be confirmed in animal experiments.
